# Sex- and Age-Related Differences in Morbidity Rates of 2009 Pandemic Influenza A H1N1 Virus of Swine Origin in Japan

**DOI:** 10.1371/journal.pone.0019409

**Published:** 2011-04-29

**Authors:** Nobuoki Eshima, Osamu Tokumaru, Shohei Hara, Kira Bacal, Seigo Korematsu, Minoru Tabata, Shigeru Karukaya, Yoshinori Yasui, Nobuhiko Okabe, Toyojiro Matsuishi

**Affiliations:** 1 Department of Biostatistics, Oita University Faculty of Medicine, Yufu City, Oita, Japan; 2 Department of Neurophysiology, Oita University Faculty of Medicine, Yufu City, Oita, Japan; 3 The Yomiuri Shimbun Osaka (Newspaper), Osaka, Japan; 4 Medical Programme Directorate, Faculty of Medical and Health Sciences, University of Auckland, Auckland, New Zealand; 5 Department of Pediatrics, Oita University Faculty of Medicine, Yufu City, Oita, Japan; 6 Department of Mathematical Sciences, Graduate School of Engineering, Osaka Prefecture University, Sakai City, Osaka, Japan; 7 Department of Pediatrics, Kurume University School of Medicine, Kurume University, Kurume City, Fukuoka, Japan; 8 Infectious Disease Surveillance Center, National Institute of Infectious Diseases, Tokyo, Japan; Massachusetts General Hospital, United States of America

## Abstract

**Background:**

The objective of the present study was to determine whether the morbidity rates of the 2009 pandemic influenza A H1N1 virus (pdmH1N1) varied by age and/or sex.

**Methods and Findings:**

Retrospective analysis of 2,024,367 cases of pdmH1N1 was performed using the national surveillance data from influenza sentinel points in Japan. The male-to-female morbidity ratios (M/F ratios) in nineteen age groups were estimated as the primary outcome. The M/F ratios for pdmH1N1 influenza were: >1 in age groups <20 years and ≥80 years (p<0.001); <1 in age groups 20–79 years (p<0.001). This data suggests that males <20 years of age may be more likely to suffer from pdmH1N1 influenza than females in the same age categories. When the infection pattern for pdmH1N1was compared with that of seasonal influenza outbreaks between 2000 and 2008, the M/F ratio for pdmH1N1 influenza was higher in ages 3–29 years and lower in ages 40–79 years. Because the present study was based on the national surveillance, it was impossible to estimate the morbidity rate for the Japanese population. It is also likely that the data did not capture asymptomatic or mild infections.

**Conclusions:**

Although exposure to the pdmH1N1 virus is assumed to be similar in both boys and girls, M/F ratios were >1 in those younger than 20 years. The subsequent reversal of the M/F ratio in the adult generation could be due to several possibilities, including: greater immunity among adult males, more asymptomatic infections among males, less reporting of illness by males, or differences in exposure to the virus and probability of visiting a clinic. These results suggest that the infection and virulence patterns of pdmH1N1 are more complex than previously considered.

## Introduction

The 2009 pandemic influenza A H1N1 virus (pdmH1N1) emerged in Mexico in April 2009 and spread rapidly worldwide [1], [2]. WHO officially declared the spreading pdmH1N1 virus to constitute a global pandemic in June 2009 [2]. In the first wave of the pdmH1N1 influenza outbreak, the rate of infection was highest in children younger than 15 years of age [3]. The high risk groups for pdmH1N1 influenza include pregnant women, children under 5 years, elderly people (over 65 years of age), and people with chronic diseases such as bronchial asthma and diabetes [1]. It has also been proposed that pregnant women have higher morbidity and mortality rates in a pandemic of pdmH1N1 influenza than in pandemics of seasonal influenza [4]. Although the clinical manifestations of pdmH1N1 infection have been generally mild to date, it is possible that the virus may reassort with human influenza viruses, potentially making it more transmissible or more pathogenic [5]–[7].

Although analysis of the transmission patterns of infectious diseases is important to help prepare for future epidemics of novel infectious diseases, it is often difficult to identify sex differences related to a disease's transmission. Differences may be related to the different longevities of males and females, to different behavior patterns, to different levels of susceptibility to the virus through the role of sex hormones on immunity [8], [9], or several other confounding factors.

Differences in sex-based transmission have been reported for several diseases. In a study of blood donors <20 years of age (i.e. before sexually transmitted diseases are likely), infection with human T-cell leukemia virus type I (HTLV-I), a retrovirus causing adult T-cell leukemia [10], was detected more frequently in males than in females [11]. This may be regarded as a sex-based difference in vertical infection of HTLV-I through breast milk, and it suggests that immunity is more mature in female infants than in male infants. Another study showed that when a mother is exposed to respiratory syncytial virus, sons are twice as likely to have respiratory syncytial virus detected as daughters [12]. In adults, humoral immune responses to vaccines are higher in females than in males, but females report more severe local and systemic adverse reactions [13], [14]. Despite these examples, however, the underlying sex-specific differences in immune development are generally ill-defined and poorly understood.

In preparing for novel infectious diseases, analysis of pdmH1N1 cases provides valuable information, as almost the entire population lacked immunity to this strain at the start of the 2009 outbreak. As a result, differences in pdmH1N1 morbidity rates with respect to sex and age could be evaluated without the confounding influence of herd immunity. In contrast, cyclical outbreaks of seasonal influenza and other common infectious diseases cannot be studied in this way, because in the usual community-acquired infectious diseases, the levels of cumulative herd immunity are likely to vary with sex and age [15], thus confounding the analysis. The aims of the present paper are twofold: to describe the dynamics of pdmH1N1 infection and to clarify the differences in morbidity rates with regard to age and sex from an epidemiological viewpoint.

## Methods

### Ethics Statement

Ethical approval and signed patient consent forms were not required for our study according to the Guideline for Epidemiological Studies established by the Ministry of Health, Labor and Welfare and the Ministry of Education, Culture, Sports, Science and Technology of Japan; all individual data were collected by law, and patients could not be identified, as all data were de-identified; i.e., stripped of personal identifiers.

### Study Population and Data Collection

Japan has an active infectious disease surveillance system. With respect to influenza, the National Institute of Infectious Disease (Tokyo, Japan) collects reports of patients with influenza-like illnesses (ILI) on a weekly basis. ILI is defined as (1) fever above 38 degrees Celsius AND (2) acute respiratory signs including one or more of: running nose, nasal congestion, sore throat or cough (identical to CDC's definition). The National Institute of Infectious Disease receives these reports from approximately 5000 sentinel points, including approximately 3000 pediatric and 2000 internal medicine clinics, i.e., approximately 10% of the medical facilities in Japan [16]. The number of the sentinel points fluctuates somewhat over time. It was 4,712 as of Dec. 31, 2008.

The first case of pdmH1N1 infection in Japan was reported on May 9, 2009. In total, 5038 cases of pdmH1N1 infection had been confirmed by real-time reverse-transcriptase-polymerase-chain-reaction (RT-PCR) by July 23, 2009 [17]. From July 24, 2009, surveillance has continued by the routine ILI surveillance system [16]. Data from these sites are based on clinical diagnosis, although part of them also use rapid influenza antigen detection tests, where the sensitivity to pdmH1N1 is comparable with that of the seasonal influenza A/H1N1 [18], [19]. Sensitivity of rapid antigen tests was 53.5–77% in Kobe and Osaka, Japan during the first month of the pandemic [19]. Almost all the patients with ILI were considered to be infected with the pdmH1N1 virus, based on analyses of isolated viruses [16]. Surveillance data from the 31^st^ week of 2009 (July 24, 2009) to the 12^th^ week of 2010 (March 28, 2010) were used in the current study. The cumulative number of pdmH1N1 cases in the study period was 2024367. Table 1 summarizes the patient data for pdmH1N1 influenza infection with respect to sex and age. Table 2 lists the influenza patient data for 2005, the year with the largest number of reported cases of influenza between 2000 and 2008.

**Table 1:** The M/F ratio and number of patients with 2009 pandemic influenza A H1N1, reported from fixed sentinel points from the 31^st^ week of 2009 through the 12^th^ week of 2010.

Age Group	Male	Female	M/F ratio¶	95%CI	p- value
**0**	11342	10044	1.072	1.043–1.101	<0.001*
**1**	26921	23222	1.101	1.082–1.121	<0.001*
**2**	32116	28560	1.068	1.051–1.085	<0.001*
**3**	44168	38639	1.085	1.070–1.100	<0.001*
**4**	58661	51523	1.090	1.077–1.103	<0.001*
**5**	70687	60816	1.110	1.098–1.122	<0.001*
**6**	74682	64690	1.098	1.086–1.109	<0.001*
**7**	76565	67106	1.085	1.073–1.096	<0.001*
**8**	75508	66064	1.085	1.074–1.097	<0.001*
**9**	73529	63305	1.100	1.088–1.112	<0.001*
**10–14**	280323	239023	1.117	1.111–1.123	<0.001*
**15–19**	98432	82778	1.133	1.123–1.144	<0.001*
**20–29**	54835	57620	0.907	0.896–0.917	<0.001*
**30–39**	37620	58505	0.626	0.617–0.634	<0.001*
**40–49**	22969	33393	0.679	0.668–0.691	<0.001*
**50–59**	10311	13960	0.749	0.730–0.768	<0.001*
**60–69**	3734	5797	0.688	0.659–0.716	<0.001*
**70–79**	2072	2508	1.010	0.951–1.068	0.745
**≥80**	987	1352	1.429	1.312–1.546	<0.001*
**Total**	1055462	968905	1.146	1.143–1.149	<0.001*

¶ : Estimated M/F ratio adjusted for subpopulations of males and females in each age group.

*: Significance with the Bonferroni's correction (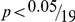).

**Table 2 pone-0019409-t002:** The M/F ratio and number of patients with seasonal influenza, reported from fixed sentinel points in 2005.

Age Group	Male	Female	M/F ratio¶	95%CI	p- value
**0**	14538	13061	1.067	1.041–1.092	<0.001*
**1**	41573	35620	1.116	1.101–1.132	<0.001*
**2**	45376	41198	1.049	1.035–1.063	<0.001*
**3**	53399	47245	1.077	1.063–1.090	<0.001*
**4**	63485	56964	1.060	1.048–1.072	<0.001*
**5**	66527	59018	1.069	1.057–1.080	<0.001*
**6**	63076	57075	1.052	1.040–1.064	<0.001*
**7**	53625	48580	1.052	1.040–1.065	<0.001*
**8**	46938	43832	1.023	1.009–1.036	0.001*
**9**	39563	36757	1.026	1.011–1.041	<0.001*
**10–14**	89129	80227	1.058	1.048–1.068	<0.001*
**15–19**	16762	15226	1.043	1.020–1.066	<0.001*
**20–29**	40034	52970	0.730	0.720–0.739	<0.001*
**30–39**	55505	85033	0.640	0.633–0.647	<0.001*
**40–49**	37546	42725	0.872	0.860–0.884	<0.001*
**50–59**	25106	29898	0.851	0.837–0.866	<0.001*
**60–69**	14803	18062	0.881	0.862–0.900	<0.001*
**70–79**	9812	10708	1.143	1.111–1.174	<0.001*
**≥80**	4556	8110	1.189	1.146–1.233	<0.001*
**Total**	781353	782309	1.048	1.045–1.051	<0.001*

¶ : Estimated M/F ratio adjusted for subpopulations of males and females in each age group.

*: Significance with the Bonferroni's correction (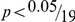).

### Statistical Analysis

The morbidity rate is the fraction of individuals in a population who develop a given illness in a particular period of time. Relative morbidity rate is defined as a ratio of morbidity rates of male and female populations of a given age group (M/F ratio).

First, to test differences in the risks of infection for males and females, a two-sided test was carried out with respect to M/F ratio for pdmH1N1 and seasonal influenza. Bonferroni's method was employed for multiple statistical comparisons in each of the 19 age groups to adjust the significance level of each comparison to a more conservative level. Next, the M/F ratios for pdmH1N1 in each of the age groups were compared to those for seasonal influenza infections in 2005.

## Results

### Infection dynamics of pdmH1N1 influenza

**Figure 1:**
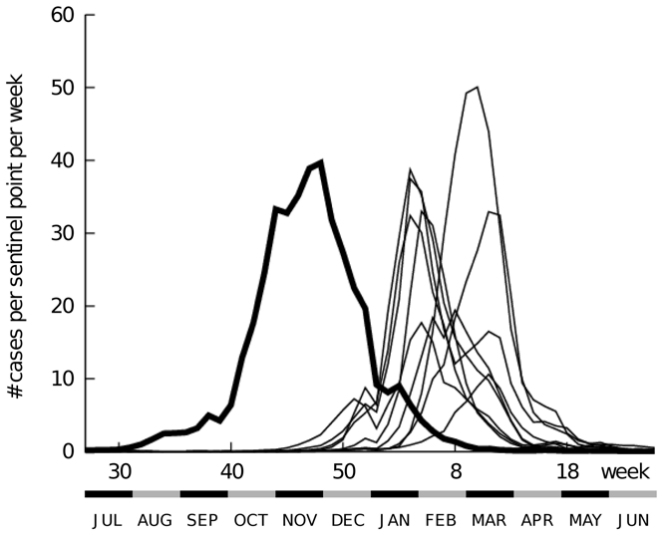
Historical view of reported cases of influenza-like illness in Japan by week of illness onset. The horizontal axis shows the year by week, beginning with the 27^th^ week of one year and going through the 26^th^ week of the next. The solid bold line indicates the number of cases per week at sentinel points in 2009. Data from cases of seasonal influenza in other years are indicated by thin lines.

Figure 1 shows the dynamics of influenza infections in Japan over the last decade. It is clear that the infection dynamics in year 2009 (bold solid line) had a different configuration from those in previous years (thin lines): The number of patients with pdmH1N1 started to increase in summer, reached its peak in late November (Nov. 23–29), and gradually decreased thereafter, whereas seasonal influenza typically had its peaks between January and March. All the cases of ILI reported from the 31^st^ week of 2009 to the 12^th^ week of 2010 were considered to be infection with the pdmH1N1 virus.

### Analysis of the seasonal influenza and pdmH1N1 case data

**Figure 2:**
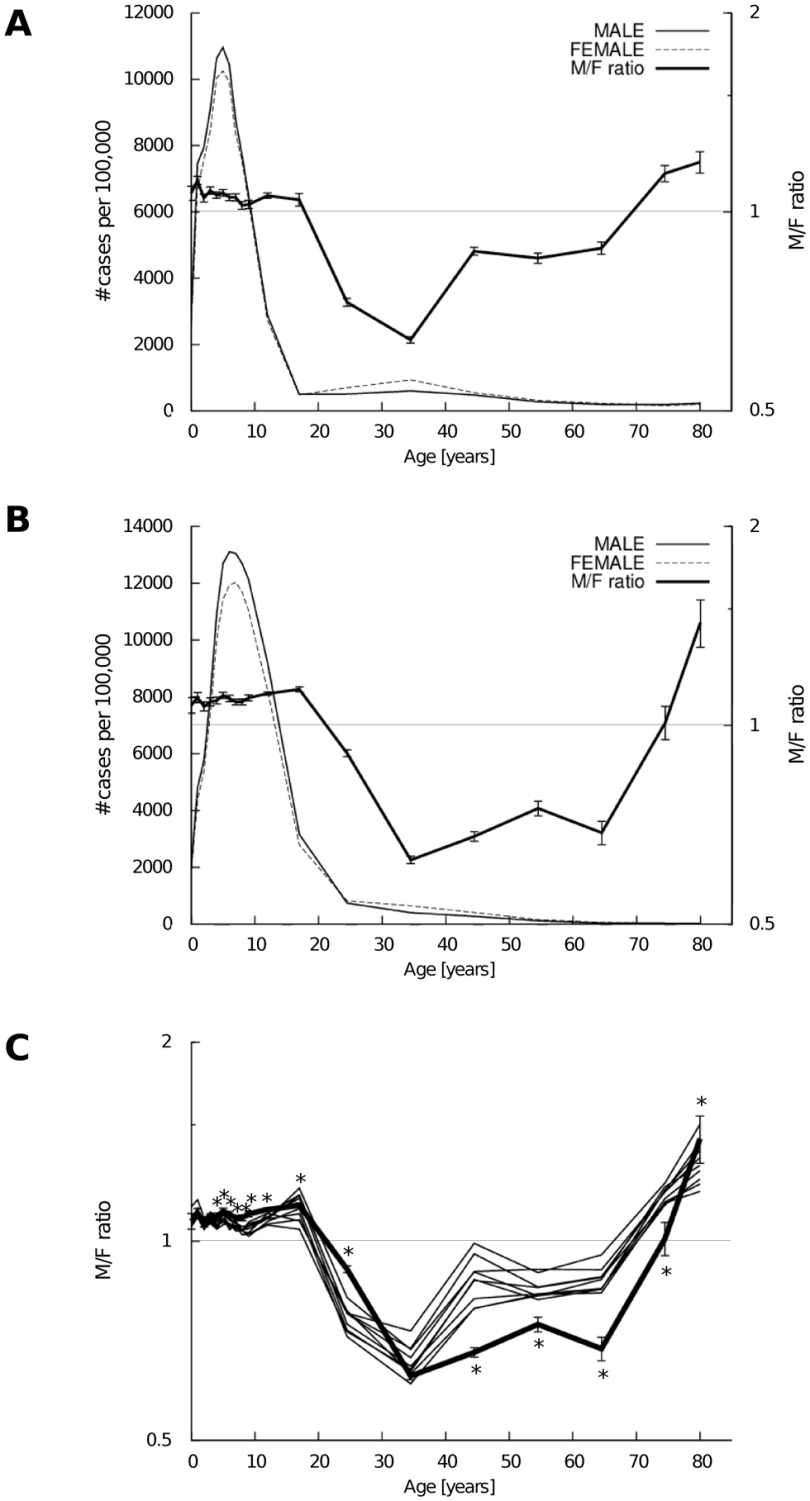
The number of cases (per 100,000 population) of influenza and the M/F ratio reported from sentinel points in Japan. The number of cases (left axis) and the M/F ratio (right axis) for seasonal influenza in 2005 (A) and for pdmH1N1 influenza in 2009 (B) reported from the sentinel points are illustrated. M/F ratios associated with pdmH1N1 (2009, solid bold line) and seasonal influenza (2000–2008, thin lines) are compared (C). 95% confidence intervals for M/F ratios are indicated by error bars. Horizontal lines indicate M/F ratio of 1. *: Significance with the Bonferroni's correction (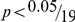).

The patient data for seasonal influenza and pdmH1N1 infections were compared with respect to sex and age. Figure 2A shows the number of seasonal influenza cases (per 100,000) in males and females as well as the M/F ratio in 2005. Two peaks in the number of cases (per 100,000) are observed: the first peak occurs in the age group of <10 years, i.e., five and four years of age for boys and girls, respectively. The second peak occurs in the age group 30 to 39 years of age with a higher number of females than males in that group.

Figure 2B shows the number of pdmH1N1 cases in males and females as well as the M/F ratio. As in figure 2A, the majority of reported patients infected with the pdmH1N1 virus are <20 years old, and there are more total cases in males than females. However, compared with seasonal influenza, the difference between the male and female subpopulations is more pronounced. The first peak is seen at age 7 years for both boys and girls. Under 20 years of age, the M/F ratio is again >1, but the M/F ratio for pdmH1N1 is higher than for seasonal influenza (figure 2C). In adults 20 to 79 years of age, the M/F ratio is <1 and the M/F ratio for pdmH1N1 is lower than the M/F ratios for seasonal influenza in people over the age of 40 years (figure 2C).

A statistical analysis of pdmH1N1 and seasonal influenza infections was next carried out. First, the morbidity rates between males and females in the 19 age groups were compared using Bonferroni's method. Tables 1 and 2 show the patient data for pdmH1N1 and seasonal influenza (2005), respectively, and the results of the statistical test are shown with p-values. For patients under 20 years of age, the M/F ratios were significantly greater than 1. This relationship was reversed for patients aged 20 to 69 years.

Next, the M/F ratios from the pdmH1N1 in the different age groups were compared with those for seasonal influenza in 2005 (table 3). As shown in table 3 and figure 2C, the M/F ratios for pdmH1N1 influenza were significantly higher in ages 4–29 years and significantly lower in ages 30–79 years. The statistical methods used in tables 1–3 are explained in the appendix S1. Supplementary data are available online as Tables S1, S2, S3, and S4.

**Table 3:** Comparison of the M/F ratios by age for 2009 pdmH1N1 with that for 2005 seasonal influenza.

Age Group	Z statistic	p- value
**0**	0.32	0.750
**1**	-1.33	0.184
**2**	1.87	0.062
**3**	0.91	0.365
**4**	3.73	<0.001*
**5**	5.53	<0.001*
**6**	6.21	<0.001*
**7**	4.36	<0.001*
**8**	8.34	<0.001*
**9**	9.40	<0.001*
**10–14**	12.46	<0.001*
**15–19**	9.12	<0.001*
**20–29**	26.85	<0.001*
**30–39**	-2.93	0.003
**40–49**	-26.42	<0.001*
**50–59**	-9.11	<0.001*
**60–69**	-12.12	<0.001*
**70–79**	-4.16	<0.001*
**≥80**	3.88	<0.001*

*: Significance with the Bonferroni's correction (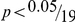).

## Discussion

### Statement of principal findings

The morbidity rates of influenza were compared through M/F ratios. Males under 20 or over 80 years of age were more likely to be identified at influenza sentinel points than females, while this relationship was reversed in adults between the ages of 20 and 79 years (figure 2B). This observation may be at least partially skewed by the decreased immunity documented in pregnancy [4]. Japanese birth rates are 62.5 and 67.9 per 1,000 females in their 20′s and 30′s, respectively [20], [21], and this constitutes a sizeable subpopulation of pregnant women, even if the Japanese birth rate is not as high as in some developing countries.

The M/F ratios for seasonal influenza also differ by sex and age (figure 2A). In the case of infants and children, where we assume that exposure to pathogens is equal and the probability of visiting sentinel points are equal for both sexes, it is speculated that the observed difference in M/F ratio may reflect a true difference in morbidity rates (figures 2A and 2B). We assume parents seek health care equally for sons and daughters, based on the similar levels of immunization for boys and girls. In 2008, male to female immunization ratios of measles-rubella combination vaccine were 0.997 (95% CI 0.993–1.000) for 1 year old, 0.991 (0.987–0.995) for preschool age (5–6 years), 0.987 (0.983–0.991) for 13 years and 0.948 (0.944–0.951) for 18 years of age [22]. Our findings in the <20 year age group could be explained if the immune responses of boys are less mature than those of girls [8], [14]. Since immunity generally increases with age, it might be possible that by adulthood, immunity in males “catches up” to that in females [8]. This would help explain the reversal of the M/F ratio in the age group >20 years.

Another possible explanation for the adult (20–79 year) M/F ratio is that there might be more subclinical cases in adult males than in adult females, suggesting a different pattern of illness between the sexes. The ratio of asymptomatic pdmH1N1 infection is estimated to be as high as 75% [23]. If men infected with pdmH1N1 are in fact less likely to develop symptoms, this ‘lack of reaction’ may be related to the fact that men are also less likely than women to develop side effects from a vaccination [14].

Another explanation could be that adult males seek medical care less frequently than females of the same age. According to a patient survey by the Japanese Ministry of Health, Labour and Welfare in 2008, the number of males who sought outpatient medical services for any reason was estimated to be 2918.5×10^3^ per day, compared with 3946.4×10^3^ per day for females [24]. Although it does not provide age-specific sex-differences in the number of patients, the difference between overall numbers of males and females is significant (p < 0.001) and would support the above hypothesis [24].

### Strength and weakness of the study

This paper is the first to discuss the morbidity rate of pdmH1N1 infection in terms of both sex and age. We speculate that infection dynamics might depend on differences in immunity as well as social activities that are influenced by age and gender [8]. As mentioned previously, the influences of sex and age on viral infections may be confounded by social roles and activities [8]. According to a WHO report, human activities are reflective of age and gender, e.g. many young children attend day-care centers or kindergartens, slightly older children go to elementary schools, and teenagers attend classes and participate in club activities at high school [8]. Adult women may be more exposed to infectious pathogens through their caretaker roles within the family, and this may lead to a higher risk of infection for adult females than for adult males [8]. Given these different types of activities, the rates of exposure to the pdmH1N1 virus could be expected to differ across age groups and by sex. If we assume equal levels of immunity in male and female adults, a significant difference in infection risks might indicate differences in exposure to the pathogen [8].

The present study had several limitations. The sentinel points are not (necessarily) chosen to represent the age distribution of Japanese population and simply encompass about 10% of clinics in Japan. It is impossible to estimate the morbidity rate for the Japanese population because the numbers of cases are reported from the selected sentinel points, not from screening the entire population [16]. However, as shown in the appendix, it is possible to estimate age-specific M/F ratios for morbidity. Since the clinical surveillance is based on individuals presenting with ILI, it does not capture asymptomatic or mild infections [16], [25]. It is also possible that our data are skewed by underreporting of illness by adult males compared with adult females.

### Strength and weakness in relation to other studies

This paper compares the morbidity rate for pdmH1N1 infection in terms of both sex and age through M/F ratios, an analysis which has not been previously performed by other papers on this topic. Wang et al estimated that the asymptomatic ratio of the pdmH1N1 infection to be 75% but did not mention any difference in morbidity rate between sexes or among age groups [23]. Current estimates of the household secondary attack rate (SAR) of pdmH1N1 range from 7.6% to 30.2% [26]–[30]. However, none of these studies mention differences between sexes or among age groups except for Odaira et al [26]. Odaira reported that household SAR in siblings was significantly higher (16.4%) than in parents (2.4%) [26]. The difference in SAR between parents and siblings was attributed to their behavior; i.e., greater contact between siblings and less satisfactory countermeasures for infection control. It would be useful, given the results of the present study, to examine household SAR with respect to sex and age.

Noymer and Garenne [31] described higher age standardized death rates in males during the 1918 influenza pandemic, along with a selection effect which had a strong and long-lasting effect on differential mortality by sex. The selection effect was that those with tuberculosis (TB) in 1918 were more likely than others to die of influenza, and tuberculosis morbidity at the time was higher in males than in females. This factor is unlikely to be an issue today due to the much lower morbidity rates for TB in current Japanese society (19.0 per 100,000 as of 2009) [32].

### Meaning of the study: possible explanations and implications for clinicians and policymakers

A major proportion of people affected by pdmH1N1 were under 20 years of age, and the M/F ratio in this group was greater than 1. We assume the rate of seeking medical care is equal for males and females in this age category. For most of these people, decisions about seeking care will be made on their behalf by parents or guardians, and there is no evidence that adults in Japan display gender-based bias when seeking care for children. Rather, documentation of similar immunization rates between boys and girls would suggest a lack of bias [22]. We therefore suggest that more males of this age developed pdmH1N1 than females.

In fact, the total number of male inpatients with pdmH1N1 was nearly twice that of female inpatients (9305 vs. 5409) [16]. Approximately 87% (12,807 out of 14,714) of the total number of inpatients with pdmH1N1 were <20 years of age, while the female population included 54 pregnant women. At first glance, the male preponderance of cases would suggest that males of any age were more susceptible to the virus. The significance of the present study is that it examines pdmH1N1 morbidity not only with respect to sex but also age. If the differences among age groups had not been investigated, the reversed relationship in patients ≥20 years of age would have been overlooked, and the true complexity of pdmH1N1infection patterns would not have been appreciated.

It is unsurprising that sex and age would both play a role in infection patterns. Sex-determined differences in susceptibility to infections are well documented; females seem to mount more vigorous immune responses and have higher resistance to bacterial and viral infections in general [33]. Vaccination studies have documented that adverse effects from vaccination against pdmH1N1 were reported in 554 males and 1,339 females, including 33 pregnant women, as of the 52^nd^ week of 2009. About 80% of these cases were <50 years of age [16]. On the other hand, more deaths related to vaccination were reported in males (72 males vs. 32 females), 95% of whom (99/104 cases) were >60 years of age [16]. Humoral immune responses to vaccines are higher in females than in males, but females report more severe local and systemic adverse reactions [14]. The mechanisms underlying sex-specific differences in immune development and sex-related differences in susceptibility to infection and vaccination efficacy remain ill-defined and poorly understood. To implement effective measures against future novel infectious disease outbreaks, differences in morbidity and mortality rates among age groups and between the sexes will need to be considered, as well as the age- and sex-specific dynamics of infection.

The clinical significance of the different M/F ratios at different ages varies. In the age group < 20 years, a relatively small difference in morbidity rate (6.5–15.8%) nevertheless translates to a large difference in the actual number of patients. By contrast, the sex-related difference is relatively large in adults (43% at 30–39 years for example) yet the difference in the actual number of patients is small.

### Unanswered questions and future research

While our results suggest possible age- and sex-specific patterns of infection in the Japanese population, they may be confounded by factors such as uneven reporting rates between the groups and should therefore be interpreted with caution. No age- and sex-specific data are currently available about illness reporting behavior of the Japanese population [24]. Further research is thus needed to help validate our findings.

As shown in figure 2C, the sex-related difference measured by M/F ratio is amplified for pdmH1N1 infection compared to seasonal influenza except in the 20–29 year age group. Questions may arise as to why the configurations of seasonal influenza and pdmH1N1 shown in figures 2A and 2B are so different and why the sex-related difference is more evident for pdmH1N1 infection than for seasonal influenza infections between 2000 to 2008 (figure 2C). While our data may be confounded by possible variation in patterns of seeking medical care among different age and sex categories, it is unlikely that such variation would be emphasized exclusively in the case of pdmH1N1. One explanation for these findings may be that almost all people lacked or had only weak immunity against the pdmH1N1 virus at the start of the 2009 outbreak, as well as the pdmH1N1 virus being more contagious than seasonal influenza viruses (although it has lower pathogenicity) [25]. Thus, the number of cases is higher in age/sex groups that have weaker immunities, more clinical infections and/or higher levels of exposure.

Another explanation would be the seasonal difference in outbreak pattern. Seasonal influenza is pandemic in winter (after New Year's Day), whereas it was in spring that pdmH1N1 started to spread in Japan, reaching its peak in late fall. This time period in Japan includes the Golden Week period (4 national holidays in a week at the beginning of May) and the Bon festival (the Buddhist festival of the dead in mid-August), and there is more national travel during these times than during winter.

It is hoped that this study will stimulate studies in other related areas of medical sciences as well as assisting in the development of strategies for the prevention and control of future epidemics.

## Supporting Information

Appendix S1 Test statistics for comparisons of morbidity ratios.
(PDF)

Table S1 The number of cases with pdmH1N1reported from the sentinel points in Japan from July 27, 2009 to March 28, 2010.
(PDF)

Table S2 Estimated Japanese population as of October 2009 (x 1,000).
(PDF)

Table S3 The number of cases with influenza reported from the sentinel points (per 100,000).
(PDF)

Table S4 M/F ratio of influenza from the sentinel points.
(PDF)
