# Supplementary material for: Sex- and Age-Related Differences in Morbidity Rates of 2009 Pandemic Influenza A H1N1 Virus of Swine Origin in Japan
Source: PLoS One. 2011 Apr 29;6(4):e19409. doi: 10.1371/journal.pone.0019409 (PMC3084848; doi:10.1371/journal.pone.0019409)
Supplement: Appendix S1 — Test statistics for comparisons of morbidity ratios. (PDF) [file pone.0019409.s001.pdf]

Appendix S1: Test statistics for comparisons of morbidity ratios

Let N_M and N_F be the subpopulations of males and females in an age group in Japanese population, i.e. fixed values, and let n_M and n_F be the random variables that describe the numbers of male and female patients with pdmH1N1 in the age group identified through ~5000 sentinel points in Japan, respectively. Since the present sampling is based on the data reported from the sentinel points, the morbidities at the current time p_M and p_F cannot be estimated from the patient data. Let π_M and π_F be the probabilities that male and female patients in the age group visit the sentinel points, respectively. Then, $\pi_M p_M$ and $\pi_F p_F$ are estimated by $\widehat{\pi_M p_M} = \frac{n_M}{N_M}$ and $\widehat{\pi_F p_F} = \frac{n_F}{N_F}$, respectively. In this paper, $\gamma = \frac{\pi_M p_M}{\pi_F p_F}$ is referred to as the M/F ratio. The ratio is the apparent M/F ratio, if $\pi_M \neq \pi_F$, and it is the true M/F ratio if $\pi_M = \pi_F$. The M/F ratio is estimated by $\widehat{\gamma} = \frac{\widehat{\pi_M p_M}}{\widehat{\pi_F p_F}} = \frac{n_M N_F}{n_F N_M}$. Since populations N_M and N_F are very large, random variables n_M and n_F can be considered to be Poisson-distributed with means $N_M \pi_M p_M$ and $N_F \pi_F p_F$, respectively. In the surveillance data, the means are sufficiently large, so from the central limit theorem, n_M and n_F are asymptotically normally distributed with variances $N_M \pi_M p_M$ and $N_F \pi_F p_F$, respectively. In order to test hypotheses with respect to the M/F ratio, the following statistic can be used:

$$Z = \frac{\widehat{\pi_M p_M} - \gamma \widehat{\pi_F p_F}}{\sqrt{\frac{\pi_M p_M}{N_M} + \gamma^2 \frac{\pi_F p_F}{N_F}}} \approx \frac{\widehat{\pi_M p_M} - \gamma \widehat{\pi_F p_F}}{\sqrt{\frac{\widehat{\pi_M p_M}}{N_M} + \widehat{\gamma}^2 \frac{\widehat{\pi_F p_F}}{N_F}}}. \quad \text{(A1)}$$

The above statistic is asymptotically distributed according to the standard normal distribution. Under the null hypothesis H_0, i.e. $\gamma = 1$, the above statistics becomes

$$Z = \frac{\widehat{\pi_M p_M} - \widehat{\pi_F p_F}}{\sqrt{\frac{\pi_M p_M}{N_M} + \frac{\pi_F p_F}{N_F}}} \approx \frac{\widehat{\pi_M p_M} - \widehat{\pi_F p_F}}{\sqrt{\frac{\widehat{\pi_M p_M}}{N_M} + \frac{\widehat{\pi_F p_F}}{N_F}}}.$$

Based on the above test statistic, we can test H_0: $\pi_M p_M = \pi_F p_F$, i.e. $\gamma = 1$ vs. H_1: $\pi_M p_M \neq \pi_F p_F$, i.e. $\gamma \neq 1$.

Next, the asymptotic confidence interval of the M/F ratio $\gamma = \frac{\pi_M p_M}{\pi_F p_F}$ is derived. For large sample, (A1) is asymptotically equal to

$$Z = \frac{\widehat{\gamma} - \gamma}{\frac{1}{\widehat{\pi_F p_F}} \sqrt{\frac{\widehat{\pi_M p_M}}{N_M} + \widehat{\gamma}^2 \frac{\widehat{\pi_F p_F}}{N_F}}} = \frac{\widehat{\gamma} - \gamma}{\sqrt{\widehat{\gamma}^2 \left(\frac{1}{n_M} + \frac{1}{n_F} \right)}}. \qquad \text{(A2)}$$

From the above standardized statistic, we have the 95% confidence interval of the M/F,

$$\widehat{\gamma} \left(1 \pm 1.96 \sqrt{\frac{1}{n_M} + \frac{1}{n_F}} \right).$$

Finally, we can test the M/F ratio differences between pdmH1N1 and Year 2005 influenza infections using the following statistic. Let $(p_{iM}, p_{iF}, \pi_{iM}, \pi_{iF}, \gamma_i)$ be parameters in an age group for pdmH1N1 ($i = 1$) and those for Year 2005 influenza ($i = 2$), which are defined above; and let (n_{iM}, n_{iF}) and (N_{iM}, N_{iF}) be the numbers of patients and subpopulations of males and females. From (A2), the estimators of M/F ratios, $\widehat{\gamma}_i = \frac{n_{iM} N_{iF}}{n_{iF} N_{iM}}$ $(i = 1, 2)$, are asymptotically normally distributed with means γ_i and variances $\sqrt{\widehat{\gamma}_i^2 \left(\frac{1}{n_{iM}} + \frac{1}{n_{iF}} \right)}$, respectively. Since $\widehat{\gamma}_i$ $(i = 1, 2)$ are independent, the following statistic is distributed according to an asymptotic normal distribution with variance 1:

$$Z_{12} = \frac{\widehat{\gamma}_1 - \widehat{\gamma}_2}{\sqrt{\widehat{\gamma}_1^2 \left(\frac{1}{n_{1M}} + \frac{1}{n_{1F}} \right) + \widehat{\gamma}_2^2 \left(\frac{1}{n_{2M}} + \frac{1}{n_{2F}} \right)}}. \qquad \text{(A3)}$$

By using the above statistic, we can test hypotheses, H_0: $\gamma_1 = \gamma_2$ vs. H_1: $\gamma_1 \neq \gamma_2$. Under the null hypothesis, the above statistic is also asymptotically normally distributed with mean 0 and variance 1.
