# Supplementary material for: Sex- and Age-Related Differences in Morbidity Rates of 2009 Pandemic Influenza A H1N1 Virus of Swine Origin in Japan
Source: PLoS One. 2011 Apr 29;6(4):e19409. doi: 10.1371/journal.pone.0019409 (PMC3084848; doi:10.1371/journal.pone.0019409)
Supplement: Table S1 — The number of cases with pdmH1N1reported from the sentinel points in Japan from July 27, 2009 to March 28, 2010. (PDF) [file pone.0019409.s002.pdf]

Table S1: The number of cases with pdmH1N1reported from the sentinel points in Japan from July 27, 2009 to March 28, 2010

age [yr]	all age	0	1	2	3	4	5	6	7	8	9	10-14	15-19	20-29	30-39	40-49	50-59	60-69	70-79	80-
**male**	1055462	11342	26921	32116	44168	58661	70687	74682	76565	75508	73529	280323	98432	54835	37620	22969	10311	3734	2072	987
**femal**	968905	10044	23222	28560	38639	51523	60816	64690	67106	66064	63305	239023	82778	57620	58505	33393	13960	5797	2508	1352
