# Supplementary material for: Sex- and Age-Related Differences in Morbidity Rates of 2009 Pandemic Influenza A H1N1 Virus of Swine Origin in Japan
Source: PLoS One. 2011 Apr 29;6(4):e19409. doi: 10.1371/journal.pone.0019409 (PMC3084848; doi:10.1371/journal.pone.0019409)
Supplement: Table S2 — Estimated Japanese population as of October 2009 (x 1,000). (PDF) [file pone.0019409.s003.pdf]

Table S2: Estimated Japanese population as of October 2009 (x 1,000)

age [yr]	all ages	0	1	2	3	4	5	6	7	8	9	10-14	15-19	20-29	30-39	40-49	50-59	60-69	70-79	80-
**total**	127510	1078	1092	1084	1072	1050	1088	1111	1145	1160	1180	5949	6079	14417	18306	16407	16872	17798	12722	7899
**male**	62130	553	560	556	550	536	557	570	587	595	606	3047	3113	7382	9279	8256	8380	8609	5725	2671
**female**	65380	525	532	528	522	513	532	542	558	565	574	2902	2967	7034	9027	8152	8493	9189	6997	5229
