# Supplementary material for: Sex- and Age-Related Differences in Morbidity Rates of 2009 Pandemic Influenza A H1N1 Virus of Swine Origin in Japan
Source: PLoS One. 2011 Apr 29;6(4):e19409. doi: 10.1371/journal.pone.0019409 (PMC3084848; doi:10.1371/journal.pone.0019409)
Supplement: Table S3 — The number of cases with influenza reported from the sentinel points (per 100,000). (PDF) [file pone.0019409.s004.pdf]

Table S3: The number of cases with influenza reported from the sentinel points (per 100,000)

age [yr]		all ages	0	1	2	3	4	5	6	7	8	9	10-14	15-19	20-29	30-39	40-49	50-59	60-69	70-79	80-
**male**	**2000**	633.5	1282.6	3532.7	3849.0	4282.5	4825.9	5238.5	4864.8	4767.5	3550.8	2539.6	1539.3	576.8	235.2	276.7	195.0	115.7	104.7	94.5	75.4
	**2001**	250.6	601.7	1608.2	1715.1	1844.1	2009.5	1968.4	1715.7	1405.8	1211.5	1012.9	634.1	222.0	117.1	124.7	72.7	36.3	29.5	27.6	27.3
	**2002**	611.1	1582.0	4003.3	4345.4	4704.8	4544.5	4475.6	3783.1	3672.4	3220.4	3095.9	2198.0	543.5	211.4	231.6	144.6	59.5	44.8	39.1	36.7
	**2003**	945.0	2666.1	6095.5	6211.3	6712.2	7357.2	6499.7	6006.2	5702.5	5311.0	4742.2	3348.1	1020.3	330.0	309.3	231.6	120.3	96.7	93.9	109.7
	**2004**	625.6	1641.5	4233.8	3826.7	4055.2	4433.3	3837.0	2951.0	2664.8	2590.9	2769.6	2538.1	1032.0	280.0	234.0	179.8	84.0	66.1	69.7	82.9
	**2005**	1253.2	2693.9	7453.0	7941.5	9071.5	10633.9	10954.9	10438.0	8778.0	7719.0	6529.2	2893.2	496.9	503.4	594.5	473.3	265.4	192.3	185.3	224.0
	**2006**	732.7	1730.8	4594.4	4665.0	5018.5	5800.0	6052.8	5332.4	5009.2	4232.8	3652.6	2208.3	671.7	295.8	300.4	240.2	123.2	83.5	83.4	100.4
	**2007**	994.7	1767.8	4746.4	5198.3	6028.6	7220.8	7521.0	7492.3	7448.4	7002.8	6103.1	4134.8	1010.5	371.9	335.9	267.2	130.9	81.2	82.6	95.3
	**2008**	509.3	1140.0	2828.7	2963.0	3688.5	4527.4	5120.6	4589.3	3897.8	3223.7	2882.6	1464.3	360.5	228.1	224.9	152.4	58.6	30.4	26.4	27.9
	**mean (00-08)**	728.5	1664.4	4324.0	4513.0	5035.9	5693.4	5741.7	5228.5	4810.9	4227.0	3696.1	2313.0	655.6	281.9	294.0	216.6	110.7	80.9	78.6	87.5
	**pdmH1N1**	1698.8	2051.0	4807.3	5776.3	8030.5	10944.2	12690.7	13102.1	13043.4	12690.4	12133.5	9200.0	3162.0	742.8	405.4	278.2	123.0	43.4	36.2	37.0
**female**	**2000**	577.5	1187.9	3178.2	3690.0	4000.3	4625.1	4836.3	4591.3	4516.6	3427.9	2466.8	1396.3	490.9	302.2	378.1	196.4	129.0	109.8	77.2	50.2
	**2001**	229.8	533.2	1392.6	1581.2	1668.4	1880.1	1788.2	1572.4	1355.9	1164.1	970.0	586.1	189.3	150.4	181.2	76.0	42.5	33.3	23.2	19.3
	**2002**	562.4	1485.4	3646.9	4072.4	4322.6	4315.8	4077.8	3588.8	3443.6	3102.5	3043.1	2051.2	485.0	288.1	364.4	165.2	72.9	52.9	32.7	26.7
	**2003**	878.0	2531.5	5613.1	5963.6	6254.1	6951.7	6010.3	5768.0	5391.9	5107.8	4502.5	3097.4	927.0	460.3	508.1	292.5	145.4	110.4	77.7	84.2
	**2004**	581.7	1496.9	3870.0	3658.0	3811.8	4250.4	3622.3	2771.0	2571.7	2497.7	2590.3	2302.5	961.5	356.8	371.5	227.3	101.2	79.1	60.8	67.9
	**2005**	1195.8	2525.7	6676.5	7569.3	8424.6	10031.2	10251.5	9918.4	8340.7	7547.0	6363.5	2734.4	476.6	690.0	928.9	542.6	311.7	218.2	162.1	188.4
	**2006**	677.7	1596.0	4093.8	4399.4	4664.4	5535.9	5612.0	5071.2	4785.4	4080.7	3559.3	2079.8	623.2	380.5	450.9	266.9	135.8	92.2	70.2	75.0
	**2007**	904.7	1625.0	4376.1	4825.6	5581.1	6762.4	6826.7	6926.6	6993.6	6653.3	5781.3	3796.0	870.0	496.4	519.2	326.3	157.3	95.8	72.8	76.8
	**2008**	459.7	1066.2	2601.9	2775.5	3331.1	4165.5	4644.5	4266.7	3637.6	3099.3	2711.5	1353.0	299.7	277.6	328.0	169.7	70.9	35.9	22.7	21.8
	**mean (00-08)**	674.5	1549.0	3923.2	4272.5	4665.1	5378.6	5295.5	4929.0	4553.6	4075.7	3548.3	2140.8	588.0	373.0	450.5	250.5	129.8	91.9	67.0	69.1
	**pdmH1N1**	1482.0	1913.1	4365.0	5409.1	7402.1	10043.5	11431.6	11935.4	12026.2	11692.7	11028.7	8236.5	2790.0	819.2	648.1	409.6	164.4	63.1	35.8	25.9
