# Supplementary material for: Sex- and Age-Related Differences in Morbidity Rates of 2009 Pandemic Influenza A H1N1 Virus of Swine Origin in Japan
Source: PLoS One. 2011 Apr 29;6(4):e19409. doi: 10.1371/journal.pone.0019409 (PMC3084848; doi:10.1371/journal.pone.0019409)
Supplement: Table S4 — M/F ratio of influenza from the sentinel points. (PDF) [file pone.0019409.s005.pdf]

Table S4: M/F ratio

age [yr]	all ages	0	1	2	3	4	5	6	7	8	9	10-14	15-19	20-29	30-39	40-49	50-59	60-69	70-79	80-
**2000**	1.097	1.080	1.112	1.043	1.071	1.043	1.083	1.060	1.056	1.036	1.030	1.102	1.175	0.778	0.732	0.993	0.897	0.954	1.225	1.502
**2001**	1.091	1.128	1.155	1.085	1.105	1.069	1.101	1.091	1.037	1.041	1.044	1.082	1.173	0.778	0.688	0.957	0.853	0.884	1.190	1.412
**2002**	1.087	1.065	1.098	1.067	1.088	1.053	1.098	1.054	1.066	1.038	1.017	1.072	1.121	0.734	0.636	0.875	0.816	0.846	1.196	1.372
**2003**	1.076	1.053	1.086	1.042	1.073	1.058	1.081	1.041	1.058	1.040	1.053	1.081	1.101	0.717	0.609	0.792	0.827	0.875	1.209	1.302
**2004**	1.076	1.097	1.094	1.046	1.064	1.043	1.059	1.065	1.036	1.037	1.069	1.102	1.073	0.785	0.630	0.791	0.830	0.835	1.147	1.220
**2005**	1.048	1.067	1.116	1.049	1.077	1.060	1.069	1.052	1.052	1.023	1.026	1.058	1.043	0.730	0.640	0.872	0.851	0.881	1.143	1.189
**2006**	1.081	1.084	1.122	1.060	1.076	1.048	1.079	1.052	1.047	1.037	1.026	1.062	1.078	0.777	0.666	0.900	0.907	0.905	1.187	1.339
**2007**	1.099	1.088	1.085	1.077	1.080	1.068	1.102	1.082	1.065	1.053	1.056	1.089	1.162	0.749	0.647	0.819	0.832	0.848	1.135	1.242
**2008**	1.108	1.069	1.087	1.068	1.107	1.087	1.103	1.076	1.072	1.040	1.063	1.082	1.203	0.822	0.686	0.898	0.827	0.848	1.160	1.279
**mean (00-08)**	1.080	1.075	1.102	1.056	1.079	1.059	1.084	1.061	1.057	1.037	1.042	1.080	1.115	0.756	0.653	0.865	0.853	0.881	1.174	1.266
**pdmH1N1**	1.146	1.072	1.101	1.068	1.085	1.090	1.110	1.098	1.085	1.085	1.100	1.117	1.133	0.907	0.626	0.679	0.749	0.688	1.010	1.429
